# Monthly Summary

**Published:** 1869-02

**Authors:** 


### MONTHLY SUMMARY.

*Fracture of the Nasal Bones and Right Superior Maxilla, with Displacement of the Ball of the Eye.*—Von Langenbeck relates in the *Archiv. f. Opthalmolog.*, xiii., the case of an officer of a railroad, who in consequence of an injury inflicted on him by a locomotive engine, had the bones of his nose entirely crushed in; at the same time a fracture was produced of the orbitary process of the right upper jaw; an opening was made in the right antrum, with laceration of the eyelids and right cheek. The eye had been forced through an opening in the floor of the orbit, of a finger's breadth, into the right antrum, in such a manner that the axis of the eye was directed perpendicularly upwards. By separating, as far as possible. the edges of the fracture in the orbital plate, the globe of the eye was replaced in the orbit with a continuance of the power of vision. After the healing of the wounds in the eyelids, there was an inability to raise the upper one, and in consequence the sight was interfered with. By two plastic operations, this difficulty was removed to a sufficient extent to enable the patient to see with the injured organ. After a time this became attacked with superative *keratitis*, with wasting of the entire globe.—*Centralblatt f.d. Medicinisch Wissenschenaft, and Amer. Jour. Med. Sci.*

*Apparent Exercise of Volition during Anœsthesia Complete in all Other Respects.*—Called March 3d, 1868, to M——, æt. 10 years; female, nervous temperament fully marked; suffering with odontalgia; was very unwilling to allow extraction of the diseased tooth and closed the jaws so strenuously as to successfully resist every effort to part them. At the solicitation of the parents, I subjected her to the influence of chloroform, during the administration of which, she had to be forcibly held upon the couch, so great was her repugnance to extraction, even when assured it would be painless. I repeated my effort to separate the jaws, while anæsthesia was yet imperfect, but opposing volition was still exercised and baffled the attempts. During this period she opened the mouth and permitted an examination of the tooth, under the assurance that I would not attempt its extraction. When every other evidence of full and complete anæsthesia was present, I still found the jaws so strongly held together as to necessitate leverage in opening the mouth for admission of the extracting forceps. The resistance was not such as we find in tonic spasms, but appeared to be the result of voluntary effort, and never yielded, though the anæsthetic was used until the condition of the pupils and pulse forbade its further employment. There was not the least sign of any spasm or rigidity of any other muscles of the system, but on the contrary, the usual relaxation.—W. H. SHEPHERD, M. D., *in Rich. & Louis. Med. Jour.*

---

*The Structure of the Tactile Corpuscles Demonstrated.*—M. Rouget (*London Med. Mirror,*) believes that he has succeeded in demonstrating the structure of the tactile corpuscles; from frequent observations he is warranted in saying that the nerve-fibres actually enter the substance of the cone-like corpuscle instead of being merely coiled round it, as was formerly believed. His specimens are prepared by soaking them for some time in acidulated water, and then acting on them with strong nitric acid, which stains the nerve fibres, but not the other tissues.—*Medical Record.*

---

*Amaurosis from Neuralgia of Dental Nerves.*— In Zehender's *Klin. Monatsbl. f. Augenheilk,* Dr. Alexander describes a case in which for five weeks the patient had experienced a constantly increasing defect of vision in both eyes, without any change in the appearance of the organs excepting some symptoms of hyperæmia of the pupil, which the ordinary antiphlogistic treatment failed to remove. The patient suffered from neuralgia of the dental nerves connected with carious teeth; upon the extraction of the latter the dental suffering ceased, and soon afterwards the loss of vision was removed; at first on the side corresponding with that from which the decayed teeth were extracted, but after the lapse of a few days the sight of both eyes became fully restored.—*Centralblatt f.d. Medicinisch. Wissenschaften*, June, 1868. D. F. C.

---

*Large Salivary Calculus Removed from Wharton's Duct of Right Submaxillary Gland.*—By JOHN L. FIRESTONE, M. D., of Salem, Ohio.

A farmer, æt. fifty, called on me with a tumor in the floor of the mouth, under the right side of the tongue, which had been noticed a "good while," as it interfered somewhat with mastication, though not with deglutition.

On examination a hard tumor was felt in the place designated, apparently about the size of a hulled walnut, and on the floor of the mouth Wharton's duct whichwas seen unusually open. A probe was easily introduced, and immediately below the surface struck a hard, rough calculus. Introduced a narrow-bladed bistoury and opened the sac parallel with the tongue, and with the scoop end of a director, I removed a calculus 14 lines long, 8 broad, and 6 deep, and 1½ drachm in weight. It was yellowish-white; oval, with flattened sides; and was composed of "phosphate and carbonate of lime, held together by animal matter."

This salivary calculus is probably of unusual size. Rokitansky says, referring to these formations, that they vary in size, "from a millet seed to a pea, to even that of a hazel-nut."—*American Jour. of Med. Sciences*

---

*Removal of a Portion of the Inferior Maxilla for a Fibro Cartilaginous Tumor.*—Dr. W. H. Davies, Assistant Surgeon to California State Woman's Hospital, relates (*California Medical Gazette*, September, 1868) an interesting case of this:—

"I. S., æt. 10, was brought by his parents to consult me about a tumor involving a portion of the right half of the inferior maxilla. The tumor had been correctly pronounced by several surgeons to be fibro cartilaginous; but the point to be determined was, the operation necessary for its removal—whether the tumor could be removed without taking with it any portion of the maxilla. I advised the removal of the tumor,and a portion of the maxilla, to which they readily agreed; and two days after, the patient being under the influence of chloroform, I proceeded to perform the operation advised by Mr. Syme, by making an incision downward from the angle of the mouth, thence along the tumor to the extent of about six inches. Having carefully separated the gap from the tumor, I extracted two teeth, so as to permit of the more easy division of the bone, and having partly sawed through the jaw, completed the division with strong cutting pliers, and having turned the bone outwards and separated its connection with the muscles and mucous membrane of the mouth completed the operation.

"I then lightly plugged the cavity with lint, brought the ends of the divided maxilla as nearly in the line as possible, by strong silver wire attached to the teeth, and carefully approximated the edges of the wound by silver sutures; applied a bandage, which I ordered to be kept constantly wet, over the whole line of incision. The patient was fed by means of a tube and nutrient enemata. The sutures were removed on the sixth day, and by the tenth I had the satisfaction of seeing a complete union of the entire wound. On the twenty-first day I allowed him to go home, and I did not again see him for about five weeks, when I found the space between the ends of the bone nearly filled up by a tough semi-tendinous structure, and the whole jaw tolerably firm. I did not see him again for three months when he presented himself, a perfect picture of good health, the side operated on being of rather a better shape than the sound one; but, on opening his mouth I was greatly surprised to find in the centre of the new structure a perfectly formed tooth. For this I can by no means account. The portion of the maxilla was completely removed not even a piece of periosteum being left. Whence then the tooth? I have not been able thus far to hear of any similar case. Up to the present time, the boy remains perfectly well, using the right as freely as the left side, and employs the new tooth as a means of mastication.—*Amer. Jour. of Med. Sciences.*

